# Do routinely measured risk factors for obesity explain the sex gap in its prevalence? Observations from Saudi Arabia

**DOI:** 10.1186/s12889-015-1608-6

**Published:** 2015-03-17

**Authors:** Fatima Garawi, George B Ploubidis, Karen Devries, Nasser Al-Hamdan, Ricardo Uauy

**Affiliations:** Department of Population Health, Faculty of Epidemiology and Population Health, London School of Hygiene and Tropical Medicine, London, UK; Department of Global Health and Development, Faculty of Public Health and Policy, London School of Hygiene and Tropical Medicine, London, UK; King Fahad Medical City, King Saud Bin Abdulaziz University for Health Sciences, Riyadh, Saudi Arabia

**Keywords:** Obesity, Sex differences, Gender differences, Mediation analysis, Health inequalities, Social epidemiology, Social determinants of health, Saudi Arabia, GCC

## Abstract

**Background:**

The prevalence of adult obesity is higher in women than men in most countries. However, the pathways that link female sex with excess obesity are still not fully understood. We examine whether socioeconomic and behavioural factors may mediate the association between sex and obesity in the Saudi Arabian setting where there is female excess in obesity.

**Methods:**

We performed a mediation analysis using a cross-sectional, national household survey from Saudi Arabia with 4758 participants (51% female). A series of multivariable regression models were fitted to test if socioeconomic position, physical activity, sedentary behaviour, diet, and smoking mediate the association between sex and obesity (BMI >=30). The findings were confirmed using causal mediation analysis.

**Results:**

Women in this sample were roughly twice as likely as men to be obese (crude OR 1.9; 95% CI 1.6-2.3). The odds ratio remained significantly higher for women compared to men in models testing for mediation (OR range 1.95–2.06). Our data suggest that indicators of socio-economic position, physical activity, sedentary behaviour, diet, and smoking do not mediate the sex differences in obesity.

**Conclusions:**

Our analysis shows that most commonly measured risk factors for obesity do not explain the sex differences in its prevalence in the Saudi context. Further research is needed to understand what might explain the female excess in obesity prevalence. We discuss how data related to the lived experience of Saudi men and women may tap into underlying mechanisms by which the sex difference in obesity prevalence are produced.

**Electronic supplementary material:**

The online version of this article (doi:10.1186/s12889-015-1608-6) contains supplementary material, which is available to authorized users.

## Background

The prevalence of adult obesity is higher in women than men in most countries [1]. While women’s reproductive role is associated with the risk of obesity [2], studies have also shown that the magnitude of the sex differences in obesity prevalence varies vastly across countries [3-5]. This suggests that factors beyond women’s biology may also be at play and implicates the context in which individuals live. Economic indicators and gender inequality have been reported to be associated with the sex gap in obesity based on multi-country, ecological studies [4-7]. Moreover, sex-specific patterning in socio-economic position (SEP), a known predictor of obesity, and behavioural variables is well documented [8-10]. In women, SEP is negatively associated with obesity in high-income countries and positively in low-income countries [8]. Associations between SEP and obesity in men are mostly non-significant in higher-income countries but are positively related in lower-income countries [8]. Physical inactivity is another risk factor for obesity and activity levels are generally lower in females than males, with reported sex differences across all age groups [11-13]. However, while studies have identified sex-specific patterning in the predictors of obesity, few individual-level studies have formally assessed whether these routinely measured risk factors for obesity explain its sex gap.

In the present study we undertake such an analysis in the context of Saudi Arabia, a country where there is a female excess in obesity. Sex differences in obesity prevalence among adults in Saudi Arabia have been reported as early as the 1980s. Between 1989 and 2005, five national surveys [14-17] were conducted to measure the non-communicable disease (NCD) burden in the country and in all surveys the prevalence of obesity was significantly higher among women than men, ranging from 24.9% (females) and 15.6% (males) in 1989 [18] to 43.8% (females) and 28.3% (males) in 2005 [17]. Overall prevalence of obesity in adults increased from 20.8% in the early 90s to 36.2% in 2005, a 74% relative rise in just over a decade. Obesity is a major risk factor of cardiovascular diseases (CVD), which account for 27% of all deaths in Saudi Arabia [19]. Obesity is also a key driver of the diabetes epidemic which affects over 20% of the adult (over 25) Saudi population [17]. This has major financial consequences for the country, with 23% of all health care expenditure going toward the treatment of diabetes [19,20]. It has been reported that even a modest reduction of 5% in the prevalence of obesity in Saudi Arabia could prevent half a million cases of diabetes by 2030 [21]. Maternal obesity is also linked to numerous pregnancy complications such as pregnancy hypertension and gestational diabetes [22] and to adverse outcomes for the foetus, including congenital birth defects, macrosomia, intrauterine death, preterm birth, and adult disease [23]. Thus there is a need to understand the mechanisms that produce, in some countries, the female excess in obesity.

The prevalence of obesity in the Gulf region is among the highest in the world [21], and across the other five countries of the Gulf Cooperation Council (Bahrain, Kuwait, Oman, Qatar, and UAE) women’s obesity levels are nearly double that of men’s [24]. In contrast, in other high-income countries, such as Germany or Norway, there are no large sex differences in the prevalence of obesity at the national level [24]. One key aspect that differentiates the Gulf states from other high-income countries is that they also rank high on gender inequality [25]. To what extent this may impact behavioural and socioeconomic determinants of obesity is unclear, but studies have suggested that restrictions on women’s freedom of movement in the Gulf countries may contribute to their high levels of obesity [26]. Therefore, data from the Gulf region might provide novel insights into the pathways leading to the female excess in the prevalence of obesity. Saudi Arabia, the largest and most populous country in the region, is a particularly relevant setting because not only is there a significant sex gap in the prevalence of obesity but it is also a country in which the day-to-day lived experience of men and women contrasts starkly due to statutory sex-segregation.

Thus, in this study we attempt to elucidate the underlying mechanisms that may produce the excess in obesity prevalence among Saudi women by examining whether routinely measured risk factors for obesity, such as socio-economic and health-related behaviour variables, mediate the association between sex and obesity.

## Methods

### Sample overview

The survey used as the basis for the current analysis is a national, cross-sectional household survey conducted between August 2004 and 2005 by the Ministry of Health (MOH) of Saudi Arabia to estimate the prevalence of risk factors of non-communicable diseases (NCDs) in the population [17]. Permission to use the data was obtained from the Saudi MOH. The sampling methods and data collection were based on the WHO STEPwise approach to chronic disease risk factors surveillance [27]. A multistage stratified random sampling technique was used to recruit male and female Saudis aged between 15–64 years from the 5 regions (Central, East, West, North, South) of the country. This survey only included Saudi nationals, 90% of whom are ethnically Arab [28]. The design aimed to ensure that the total number of households selected from each region was proportional to the region’s population. A detailed description of the sampling methods has been reported elsewhere [17,29]. A sample size of 5000 participants was planned and a total of 4883 respondents provided information (98% response rate) [17]. To ensure a high response rate, participants were contacted through their primary health care center, visited at their homes and reminded by repeated telephone calls as well as at schools, mosques and social clubs to participate in the survey. Crucial demographic variables were missing for 125 records and thus were excluded. The current analysis is based on 4758 participants (97% of responders).

### Data collection

Data in this survey were collected using the STEPS Instrument for NCD Risk Factors. As described in the survey report [17], the questionnaire was translated into Arabic and then back translated to English to ensure accuracy of translation. The Arabic instrument was pretested for wording and understanding of the questions. Items which required adaptation to the local environment were modified accordingly [17]. Trained personnel conducted all physical examinations, including measuring weight and height of participants and conducting personal interviews with the participants to obtain self-reported data on socio-demographics, physical activity, dietary and smoking habits [17,29].

### Outcome variable

Obesity, defined based on Body Mass Index (BMI ≥ 30, for both sexes) and coded as a binary variable (obese/not obese), served as the outcome variable in the regression model. Objectively measured body weight and height were used to calculate BMI.

### Exposure variable

Sex, reported as male or female, served as the main variable of interest.

### Covariates

Obesity is known to increase with age, therefore age modelled as five 10-year age groups (15–24, 25–34, 35–44, 45–54, 55–64 years) was adjusted for in the analysis. We also adjusted for region as obesity prevalence varies across the 5 regions.

### Potential mediators of the sex-obesity association

Several variables shown in the literature to be known predictors of obesity were explored as potential mediators of the sex-obesity association, including indicators of socio-economic position (education, household income), physical activity, sedentary behaviour, diet and smoking. Educational level was classified into 3 levels for the analysis (primary school or less, secondary/vocational, college/postgraduate degree.) Estimated household income per month was classified into less than 5000 SAR, between 5000 and 10000 SAR, and greater than 10000 SAR (1USD=3.75 SAR). Current smoking was defined as a binary variable, with smokers defined as those currently consuming any tobacco product (cigarettes, pipes, cigars, shisha). Dietary data comprised combined number of fruit and vegetable servings per day [30]. The data were zero-inflated, and therefore grouped into 3 categories for the analysis (one serving or less, between 1 and 5 servings, 5 servings or more.) Similarly, self-reported time spent sedentary per day was zero-inflated and categorized into 3-hr intervals, 0–180 minutes, 180–360 minutes, and 360+ minutes.

The survey used the 16 item Global Physical Activity Questionnaire (GPAQ) to obtain data on physical activity participation in 3 domains (work, transport, recreational) [17,31]. Since a substantial proportion of participants reported no activity in any domain, we categorized physical activity variables into 3 levels based on whether they met the following criteria defined in the GPAQ analysis guidelines [32] (activity related energy expenditure was calculated in terms of MET-minutes; one MET (Metabolic Equivalent of Task) is defined as the energy cost of resting and is approximately an expenditure of 1 kcal/kg/hour) [33]: (1) *High level of physical activity:* (i) vigorous-intensity activity on at least 3 days achieving a minimum of at least 1500 MET-minutes/week or (ii) daily activity of any combination of walking, moderate-or vigorous-intensity activities achieving a minimum of at least 3000 MET-minutes per week.

(2) *Moderate level of physical activity:* (i) 3 or more days of vigorous-intensity activity of at least 20 minutes per day or (ii) 5 or more days of moderate-intensity activity or walking of at least 30 minutes per day or (iii) 5 or more days of any combination of walking, moderate-or vigorous-intensity activities achieving a minimum of at least 600 MET-minutes per week. (3) *Low level of physical activity:* participants not meeting either of the above criteria.

### Statistical analysis

**Figure 1:**
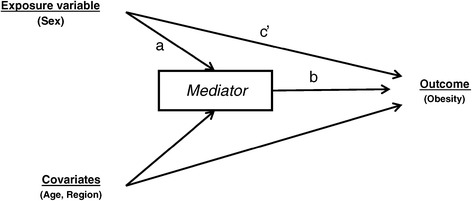
**Causal Diagram: Sex, the exposure variable, has both a direct effect on obesity (path c’) and an indirect effect on obesity (path a and b) via the mediators (SEP, physical activity, sedentary behaviour, smoking, diet).** Age and region are covariates.

The aim of our analysis was to examine the underlying process by which socio-economic and health behavior variables may mediate the relationship between obesity and sex. Figure 1 shows the causal diagram used in our analysis. Mediation was first assessed informally by fitting a series of models and observing the relative change in the magnitude of the parameter that captures the association between the exposure variable (i.e., sex) and obesity. To that end, we fitted logistic regression models to test the association between obesity status (obese/not obese) and sex (female/male), adjusted for age and region. We compared the change in magnitude of the parameter for sex when blocks of mediators were entered into the model. We first entered socio-economic variables (education and household income) as a block, followed by level of physical activity, time spent sedentary, smoking, and combined fruit/vegetable servings per day. The final model included all variables (full model). We confirmed the results of the informal analysis with causal mediation analysis for nonlinear models as implemented by the Stata command *medeff* [34,35]. The *medeff* function involves two steps. First, two regression models are fitted, one in which the mediator is regressed on the exposure variable adjusted for covariates, and a second in which the outcome is regressed on the exposure and mediator variable, adjusted for covariates. Predictions from these models are then used within a Monte-Carlo framework to calculate estimates for total, indirect and direct effects [36]. This process decomposes the total effect of sex on obesity into a direct and indirect (i.e., mediated) effect and produces a proportion of the total effect that is mediated (ratio of indirect/total effect.) Because *medeff* can only handle binary and continuous mediators, we used the continuous version of the mediator where possible (age and education) or reclassified the mediator as a binary variable (all other variables.) The cluster sampling design of the survey was taken into account during analysis by using Stata’s *svy* commands [37].

## Results

Females accounted for 51% (2418 out of 4758) of the participants in this sample, with a mean age of 36 years (95%CI 35–36.1). The mean age for men was 37.5 years (95%CI 37–38). Participants completed on average 8 years of schooling (95%CI 7.7–8.02), with 52% having received only primary schooling or less (63% of women, 41% men). Fifty-three percent (49% males, 58% females) of all participants had a self-reported household income of less than 5000 SAR per month (1333 USD per month). By occupation, 37% identified as homemakers (100% females) and 29% as government employees (90% males.) Only 38% of the sample were in paid employment (65% males, 12% females). Prevalence of smoking was low in this sample, with only 14% of participants classified as current smokers (smoking any tobacco product), 94% of whom were males. Less than 6% (8% males, 5% females) of the sample reported consuming 5 or more servings of fruit and vegetables per day. Table 1 presents further sample characteristics.

**Table 1:** **Sample characteristics**

	**Men N (%)**	**Women N (%)**	**Total N (%)**
**Covariates**			
*Age group*			
15-24 yrs	547 (23)	529 (22)	1,076 (22)
25-34 yrs	487 (21)	643 (27)	1,130 (24)
35-44 yrs	521 (22)	646 (27)	1,167 (25)
45-54 yrs	427 (18)	414 (17)	841 (18)
55-64 yrs	358 (15)	186 (8)	544 (11)
*Region*			
Central	575 (25)	564 (23)	1,139 (24)
Eastern	351 (15)	355 (15)	706 (15)
Northern	226 (10)	229 (9)	455 (10)
Southern	494 (21)	507 (21)	1,001 (21)
Western	694 (30)	763 (32)	1,457 (30)
**Mediators**			
*Educational Level*			
Primary school or less	965 (41)	1511 (63)	2476 (52)
Secondary/vocational	1001 (43)	664 (27)	1,665 (35)
College/Post-graduate	370 (16)	238 (10)	608 (13)
*Estimated Household Income*			
Less than 5000 SAR	1123 (50)	1380 (61)	2503 (56)
5000-10000 SAR	750 (33)	579 (26)	1329 (29)
More than 10000 SAR	365 (16)	307 (14)	672 (15)
*Combined Fruit and Vegetable servings p.d.*			
less than 1 serving per day	599 (27)	763 (32)	1,362 (30)
1-< 5 serving per day	1410 (64)	1518 (63)	2,928 (64)
5 or more servings per day	185 (8)	108 (5)	293 (6)
*Level of Total Physical Activity*			
Low	1353 (60)	1713 (73)	3,066 (67)
Moderate	472 (21)	299 (13)	771 (17)
High	426 (19)	338 (14)	764 (16)
*Time spent sedentary*			
0-180 minutes per day	914 (41)	1016 (45)	1,930 (43)
180-360 minutes per day	838 (38)	729 (33)	1,567 (35)
360+ minutes per day	462 (21)	496 (22)	958 (22)
*Currently smoke tobacco products*			
Yes	577 (25)	34 (1)	611 (14)
No	1758 (75)	2382 (99)	4,140 (93)
**Outcome**			
*Obese (BMI >=30)*			
Yes	580 (26)	936 (40)	1516 (33)
No	1660 (74)	1410 (60)	3070 (67)

**Figure 2:**
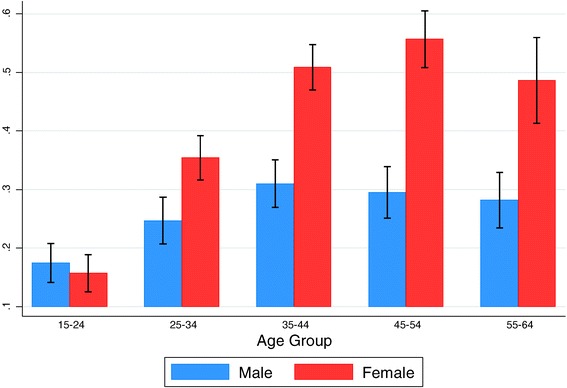
**Proportion obese in the sample stratified by sex and age group.**

**Figure 3:**
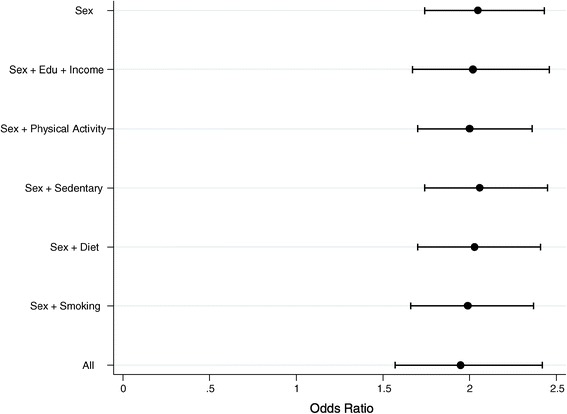
**Female-to-male odds ratio and 95% CI for the sex parameter for various models testing informally for mediation.** All models control for age and region.

Both men and women in this survey showed high levels of inactivity. On average, male and female participants spent 4.5 hours per day (95%CI 4–5 hours) sedentary. Sixty-seven percent (60% males, 73% females) of participants failed to meet the GPAQ criteria for at least moderate levels of physical activity. Ninety-three percent of participants did not engage in any vigorous physical activity in either the work or recreational domain (92% males, 93% females.) While both men and women were highly inactive, the latter had nearly twice the odds of being obese than men (crude OR=1.9; 95% CI 1.6-2.3). However, self-reported physical activity levels were not significantly associated with the obesity outcome in this sample (Log-Odds=−0.02; 95%CI–0.17, 0.13). Figure 2 shows the proportion obese stratified by age and sex. At younger ages, the proportion obese is similar for both sexes but with progressive age, obesity prevalence increases sharply in women; by age 45, women have nearly double the prevalence of men. Figure 3 and Table S1 (see Additional file 1: Table S1) present the results of the informal mediation analyses examining the association between obesity status and sex in the presence of potential mediating factors. Controlling for age and region, women were 2.05 times as likely to be obese compared to men. Entering indicators of SEP, physical activity, time spent sedentary, smoking, and diet into the model did not significantly modify women’s higher odds of obesity relative to men (Figure 3).

Table 2 presents the results of the causal mediation analysis. With the exception of time spent sedentary and fruit/vegetable intake, the exposure (sex) was significantly associated (p < 0.001) with all mediators (column 1). Being female was inversely associated with educational level, household income, physical activity, and smoking. The effect of the mediators on obesity (column 2) was positive and significant (p < 0.01) for household income and diet (fruits and vegetable consumption), but was not significant for all other mediators (p > 0.1). The effect of sex on obesity remained strongly positive and significant (p < 0.001) in all models (column 3). Overall, the variables studied did not mediate the effect of sex on obesity. The direct and total effects were near identical rounded to the nearest tenth, indicating no effect of mediation. The indirect, mediated, effects were statistically insignificant, except for household income and fruit/vegetable consumption, which were statistically significant but small in magnitude. Moreover, the effects for household income and fruit/vegetable consumption were negative and therefore inconsistent with the positive direct effect of sex on obesity. If not for their small effects, these variables may act as suppressors (increasing rather than reducing the effect of sex on obesity). To the extent that there was any effect, mediated or otherwise, the magnitude was at most one-twentieth of the total effect (last column, Table 2).

**Table 2:** **Mediated effects of sex on obesity via the selected variables**

	**Effect of sex on mediator M (path a in causal diagram)** ^**1**^	**Effect of mediator M on obesity (path b in causal diagram)** ^**2**^	**Effect of sex on obesity (path c’ in causal diagram)** ^**2**^	**Indirect Effect** ^**3**^	**Direct Effect** ^**3**^	**Total Effect** ^**3**^	**Prop. of Total Effect mediated**
**Mediator (M)**	**EST (CI)**	**EST (CI)**	**EST (CI)**	**Mean (CI)**	**Mean (CI)**	**Mean (CI)**	
Education	−3.62 (−3.99,–3.25)	−0.003 (−0.02,0.01)	0.66 (0.48,0.87)	0.002 (−0.01, 0.01)	0.136 (0.10, 0.17)	0.139 (0.11, 0.17)	0.015 (0.01,0.02)
Household Income	−0.57 (−0.73,-0.40)	0.20 (0.06, 0.34)	0.69 (0.54, 0.84)	−0.005 (−0.01,–0.001)	0.143 (0.11, 0.17)	0.137 (0.11, 0.17)	−0.04 (−0.05,0.03)
Physical Activity Levels	−0.65 (−0.91,–0.38)	−0.02 (−0.17, 0.13)	0.66 (0.46, 0.86)	0.0004 (−0.003, 0.004)	0.131 (0.10, 0.16)	0.132 (0.10, 0.16)	0.003 (0.002, 0.004)
Time spent sedentary	−0.11 (−0.32, 0.11)	0.004 (−0.15, 0.14)	0.71 (0.54, 0.88)	−0.00005 (−0.001, 0.002)	0.1469 (0.12,0.18)	0.1468 (0.12, 0.18)	−0.0003 (−0.0004,-0.0002)
Fruit/Veg consumption	−0.19 (−0.47, 0.08)	0.23 (0.0, 0.41)	0.66 (0.46, 0.86)	−0.002 (−0.005,–0.0007)	0.13 (0.10, 0.16)	0.128 (0.10, 0.16)	−0.02 (−0.02,-0.01)
Smoking	−3.24 (−3.83,–2.64)	−0.16 (−0.37, 0.06)	0.67 (0.49, 0.85)	0.007 (−0.004, 0.02)	0.139 (0.11, 0.17)	0.145 (0.12, 0.17)	0.05 (0.04,0.06)

Table provides estimates obtained from Stata’s *medeff* function. Estimates in the first three columns are obtained from the initial regression models that the function fits (described in Methods).

(1) The effect of sex on the mediator is obtained from a model regressing the mediator (as outcome) on sex and age. The coefficient and 95% confidence intervals (CI) for sex is shown in the first column. EST (estimate) is either beta coefficient from linear regression (in the case of education variable as the outcome) or log odds (all other variables. For significance at the 5% level, the 95% CI should not cross over 0.

(2) The effect of the mediator on obesity and the effect of sex on obesity are obtained from a single model regressing the outcome (obesity) on sex, the mediator, age, region, and mediator-outcome confounders. The log odds (CI) estimate for the mediator and sex are shown in the second and third column, respectively.

(3) The effects in columns 4–7 are derived by *medeff* based on the parameters in columns 1–3: Total Effect estimate is expressed as a proportion of the change in the probability of obesity. Similarly, indirect effect of sex on obesity via each of the mediators and direct effect of sex on obesity are also expressed as proportions. The direct effect is equivalent to c’ (third column) transformed on a probability scale. The last column reports the ratio of indirect effect to the total effect. A negative proportion of total effect mediated reflects inconsistent mediation.

## Discussion

Our analysis has shown that women in Saudi Arabia are nearly twice as likely to be obese than men. Given Saudi women’s restrictions on their mobility [38,39], a first line of thought suggests that the greater odds of obesity are due to women being less physically active than men or spending more time sedentary. While physical activity levels of women in this national survey were proportionately lower than that of men, the association between physical activity and obesity was not significant in this sample. Indeed, our analysis shows that, after adjustment for age, mediation by indicators of SEP, physical activity, time spent sedentary, diet, or smoking was negligible to none. Our analysis detected inconsistent mediation, since the association is positive between sex and obesity but negative along some of the indirect paths (as indicated by a negative proportion mediated) [40]. The lack of mediation may also be in part due to weak associations between the mediators under consideration and the outcome. For smoking, there was a strong negative association with obesity for men but no association for women (analysis not shown), in which case the pooled effect for both sexes tends to be non-significant. However, for the other variables the association with obesity was weak for both sexes. More empirical work may be needed to ascertain that these risk factors for obesity operate in Saudi Arabia as they do in other populations.

The finding that key predictors of obesity do not mediate the effect of sex is remarkable and suggests that in the Saudi setting other pathways need to be explored. Parity is an important variable that may partially mediate the effect of sex on obesity; however, it is missing from this analysis because it was unavailable in our dataset (although, age, for which we adjusted in the model, may be considered as a proxy for parity since number of children born is generally age-dependent.) It is known that the risk of obesity in women increases with number of children and short birth intervals [2]. Saudi women’s mean BMI ranges from 25.1 in nulliparous women to 31.7 in women with more than 4 births [41]. In 2004, the average number of children ever born to Saudi women aged 40 and above was over 5 children per woman [42]. Thus the two-fold sex gap in obesity by middle-age may be a consequence of Saudi women’s high birth rates at that time. Additionally, Saudi Arabia has low breastfeeding rates [43] which is linked with post-partum weight retention [44]. Parity may also impact on physical activity, as pregnancy has been shown to be associated with decreased physical activity [45]. Nonetheless, despite these and other potential pathways by which the sex gap may be mediated, this does not negate the principal finding that the variables considered in this analysis, which are key risk factors of obesity that are routinely measured in epidemiological surveys, do not mediate the sex-obesity association in this sample.

While physical activity and the other factors considered in this analysis did not mediate the sex gap in obesity, the *lived experience* of men and women in the sex-segregated society of Saudi Arabia may well contribute to the differences in obesity prevalence in ways beyond what is routinely measured in surveys. Foremost, the impact of women’s structural exclusion from public life on their health remains largely unexamined; it restricts the majority of women to activities within the home, where food, especially the preparation and consumption of homemade sweets, has become the currency of social exchange among women [46]. Women’s marginalization in society, in effect, foists onto them a lifestyle in which food and eating take center stage. In a culture where hospitality is a valued tradition and social courtesy obliges people not to refuse food at social gatherings lest it offend the host [46-48], excess food consumption becomes the norm. Saudi women’s structural exclusion also restricts their access to paid employment. With the majority of the female population unemployed [28], it is common practice for homemakers to remain awake all night (watching TV, using the internet, snacking, etc.) [46], and then sleep well into the early afternoon (while their husbands are at work and their children at school) their sleeping patterns in effect resembling those of night shift workers [46]. Studies among night shift workers have reported that disruption to circadian rhythm and disordered eating during night-time are associated with weight gain and greater risk of developing obesity and diabetes [49,50]. Findings from qualitative data on Saudi women [46] suggest that homemakers deliberately carve out a private space for themselves at night-time, where they are free from their obligations as wife, mother, or daughter. Technology that allows people to watch TV on demand and to use the internet at all hours of the day may have facilitated this behaviour. While these data point to possible mechanisms, epidemiological surveys are needed to confirm how prevalent this behavioural pattern may be in the Saudi population by including questions about sleeping patterns in survey instruments.

It should be noted, however, that while there is a female excess in obesity, the prevalence among Saudi men is also high (28%), although levels are comparable to the prevalence of obesity in US men in 2005 (31%) [51]. On the whole, Saudi Arabia’s built environment, with its sprawling cities that require travelling by car and the proliferation of fast food restaurant chains, are not unlike the obesogenic environment of the US and contribute to the high overall prevalence of overweight and obesity in Saudi Arabia [52,53]. The desert climate in the country further presents a barrier for outdoor activity for both men and women. But it is in a gender ideology of exclusion that Saudi men and women’s lived experience differs. Some insights on the potential impact of this on women’s health was given above; however, women’s exclusion does not occur in isolation of men, and thus further research is also needed to understand its impact on the day-to-day lived experience of Saudi men and their health.

Sex differences in obesity prevalence among those under age 35 are less pronounced than in older age groups, but due to the cross-sectional nature of this survey, it is impossible to tease out an age effect from a cohort effect. In the past 30 years, Saudi Arabia has undergone rapid social changes, especially with respect to education, such that literacy rates for those aged under 25 have reached over 97% and do not show a significant sex gap [54]. The last 12 years have also seen the country institute some gender reforms, with the provision of increased work opportunities for women. Internet and satellite TV have also been made available to the wider public [55], allowing for greater access to health information. Media exposure is also known to have a powerful effect on body image and body shape preferences [56]. Indeed, social norms around obesity have changed in Saudi Arabia and the wider Gulf region. While older generations idealized larger shapes as “a symbol of fertility and womanhood” [57], increasingly, women, as well as men, express a preference for thinner body shapes [58,59]. There have also been changes in fertility-related behaviour. In the mid-80s, Saudi women reached their peak birth rates around 25 years of age, but by the late 90s this shifted to 30–34 years, likely reflecting women’s increased educational levels and delay in marriage [60]. Fertility rates too have declined from 5.46 in 1992 to 2.91 in 2005 [61]. Thus, the narrower sex gap for those under 35 in 2005 might be due to changes in context and behaviour rather than younger age per se.

### Limitations and future work

A number of limitations must be addressed. Since this is a cross sectional study, no causal order can be inferred from any associations. We used BMI as our measure of obesity which may underestimate obesity prevalence, especially in women [62]. Self-reported data, such as physical activity, are subject to bias as respondents may misreport (e.g. recall or social desirability bias) which can result in underestimating the strength of the association with obesity [63]. Dietary variables available for this analysis were restricted to fruit and vegetable intake. Evidence exists of an inverse association between fruit and vegetable intake and adiposity [30], however future analyses would also need to consider dietary components that have more consistently been associated with obesity (e.g., energy-dense foods and beverages).

As noted, information on other possible mediators was a limiting factor. For instance, there is evidence in some populations that being married is a risk factor for obesity, although the association differs for men and women [64,65]. Thus marital status may act as a mediator, but that cannot be ascertained because it was not available in the dataset. Other factors appropriate for this setting which might help tease out the sex effect include variables capturing reproductive practices (e.g., average birth interval, breastfeeding practices, and contraceptive access and use.) As discussed, qualitative studies may also help uncover underlying social processes by which these sex differences in obesity prevalence may be produced and their findings could then inform the development of more context relevant survey instruments.

Finally, although this is a well-designed, high-quality national survey, the data are from 2005, and therefore the findings may not be current. However, despite these limitations, this study adds to the limited literature available which directly addresses the female excess in obesity prevalence.

## Conclusions

Obesity is associated with increased cardiovascular disease risk and increased healthcare costs. In women, obesity additionally poses significant risks to their reproductive health and the health of the foetus. Thus, understanding the mechanisms producing the female excess in obesity, and intervening to reduce it, addresses a public health problem of importance to women’s health. Our study showed that routinely measured risk factors for obesity do not explain the sex gap in its prevalence in Saudi Arabia. It is possible that multi-parity in older women accounts for some of the sex gap in obesity, however to our knowledge no studies in Saudi Arabia or in other countries have directly modelled this using individual-level data and so the extent to which parity, or other variables not considered in this analysis, account for the sex gap at the individual-level is unknown. Given that the magnitude of the sex gap varies vastly across countries however also suggests that reproductive factors alone are unlikely to explain the excess in female obesity. More research is needed to understand the underlying mechanisms. From our initial exploratory analysis in the Saudi setting, it appears that the local context privileges being female above other factors in determining obesity outcome. If confirmed, a broader implication then may be that the female excess in obesity prevalence in Saudi Arabia may itself be a telltale of the underlying structural imbalances between men and women that are in place. This would suggest that, beyond promoting lifestyle modifications in individuals, as advocated by the Saudi MOH [66], it may also require changing the structural position of women and men in Saudi society in order to achieve improved health outcomes for both. The impact of such changes on the sex gap in obesity prevalence, if any, may only become apparent in future epidemiological surveys.

## Additional file

Additional file 1: **BMC Supplementary materials.** Table with numerical values for Figure 3.
