# Supplementary material for: Do routinely measured risk factors for obesity explain the sex gap in its prevalence? Observations from Saudi Arabia
Source: BMC Public Health. 2015 Mar 17;15:254. doi: 10.1186/s12889-015-1608-6 (PMC4371623; doi:10.1186/s12889-015-1608-6)
Supplement: Supplementary file 1 — BMC Supplementary materials. Table with numerical values for Figure 3. [file 12889_2015_1608_MOESM1_ESM.docx]

**Do routinely measured risk factors for obesity explain the sex gap in its prevalence? Observations from Saudi Arabia**

**Fatima Garawi^1^**^§^**, George B. Ploubidis^1^, Karen Devries^2^, Nasser Al-Hamdan^3^, Ricardo Uauy^1^**

**^1^** Department of Population Health, Faculty of Epidemiology and Population Health, London School of Hygiene and Tropical Medicine, London, UK

**^2^** Department of Global Health and Development, Faculty of Public Health and Policy, London School of Hygiene and Tropical Medicine, London, UK

**^3^** King Fahad Medical City, King Saud Bin Abdulaziz University for Health Sciences, Riyadh, Saudi Arabia

**Supplementary materials**

Table S1. Female-to-male odds ratio and 95% CI for the sex variable in various models testing for mediation (informal analysis). Odds ratios for models 2-8 are displayed graphically in Figure 3 in manuscript.

| Model | Variables included in model ^(a)^ | OR | 95% CI | P-value |
| --- | --- | --- | --- | --- |
| 1 | Sex | **1.97** | 1.65 – 2.35 | <0.000 |
| 2 | Sex + region + age | **2.05** | 1.74 – 2.43 | <0.000 |
| 3 | Sex + region + age + education + income | **2.02** | 1.67 – 2.46 | <0.000 |
| 4 | Sex + region + age + physical activity | **2.00** | 1.70 – 2.37 | <0.000 |
| 5 | Sex + region + age + sedentary | **2.06** | 1.74 – 2.44 | <0.000 |
| 6 | Sex + region + age + smoking | **1.99** | 1.66 – 2.37 | <0.000 |
| 7 | Sex + region + age + diet | **2.03** | 1.70 – 2.41 | <0.000 |
| 8 | All | **1.95** | 1.57 – 2.42 | <0.000 |

1. Model 1 is crude; Model 2 adjust for the two covariates, age and region; Models 3-8 test for the potential mediators on the association between sex and obesity.
